# Intraoral transnasal approach for surgical extraction of bilateral deeply impacted mesiodens: A case report

**DOI:** 10.1002/ccr3.5037

**Published:** 2021-11-09

**Authors:** Reza Sharifi, Shervin Shafiei, Hamidreza Moslemi, Meysam Mohammadi khah

**Affiliations:** ^1^ Department of Oral and Maxillofacial Surgery Cranio Maxillofacial Research Center School of Dentistry Tehran University of Medical Sciences Tehran Iran; ^2^ Department of Oral and Maxillofacial Surgery School of Dentistry Shahid Beheshti University of Medical Sciences Tehran Iran; ^3^ Department of Oral and Maxillofacial Surgery Shahid Beheshti School of Dentistry Tehran Iran

**Keywords:** exodontics, oral surgery, supernumerary teeth

## Abstract

We describe a novel modified intranasal approach to minimize the complications of the impacted mesiodens surgical extraction. Also, it can be performed under local anesthesia with proper preoperative workup.

## Graphical abstract

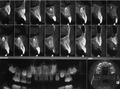

## 1 INTRODUCTION

Traditional palatal and vestibular surgical approaches for removing deeply impacted mesiodens with a crown facing the nasal floor are accompanied by significant complications. Herein, we describe a novel modified intranasal approach to minimize the complications. Also, it can be performed under local anesthesia with proper preoperative workup.

Supernumerary teeth are among the most common dental developmental anomalies. The midline of the maxilla is a common location for supernumerary teeth. Mesiodens is a supernumerary tooth located in the maxillary midline. A review of the recent literature regarding the characteristics of mesiodens revealed a prevalence rate of 0.15%–1.9% in the general population with a male/female ratio of 2.2–2.5:1.^1^, ^2^, ^3^, ^4^

Supernumerary teeth could be an isolated finding in patients and are more common in individuals with a positive family history. Moreover, some syndromes such as cleidocranial dysostosis, Gardner syndrome, Nance‐Horan syndrome, and trichorhinophalangeal syndrome are associated with supernumerary teeth.^4^ According to a popular classification, there are two types of mesiodens namely eumorphic and dysmorphic based on their similarity to central incisors in terms of shape and size. Plurality is rare in mesiodens; two mesiodens have the highest frequency of about 20%. Less than 1% of patients have three or four mesiodens.^3^, ^4^ Complications accompanied by the presence of mesiodens such as midline diastema, delayed tooth eruption, root resorption of adjacent teeth, cyst formation, and nasal eruption highlight the significance of early detection and timely surgical intervention to prevent unwanted complications and support correct dental occlusion. Surgical extraction of deeply impacted mesiodens with an inverted position invading the base of the nasal cavity can lead to serious surgical complications and morbidities such as traumatizing or injuring the nasal mucosa or adjacent structures, that is, the roots of the adjacent permanent teeth.^3^, ^5^, ^6^ In such occasions, an efficient surgical protocol with maximum patient comfort, minimal complications, and no harm to the adjacent structures is imperative. Herein, we describe a novel modified intranasal approach to minimize the complications. Also, it can be performed under local anesthesia with proper preoperative workup.

## 2 REPORT OF THE CASE

**FIGURE 1:**
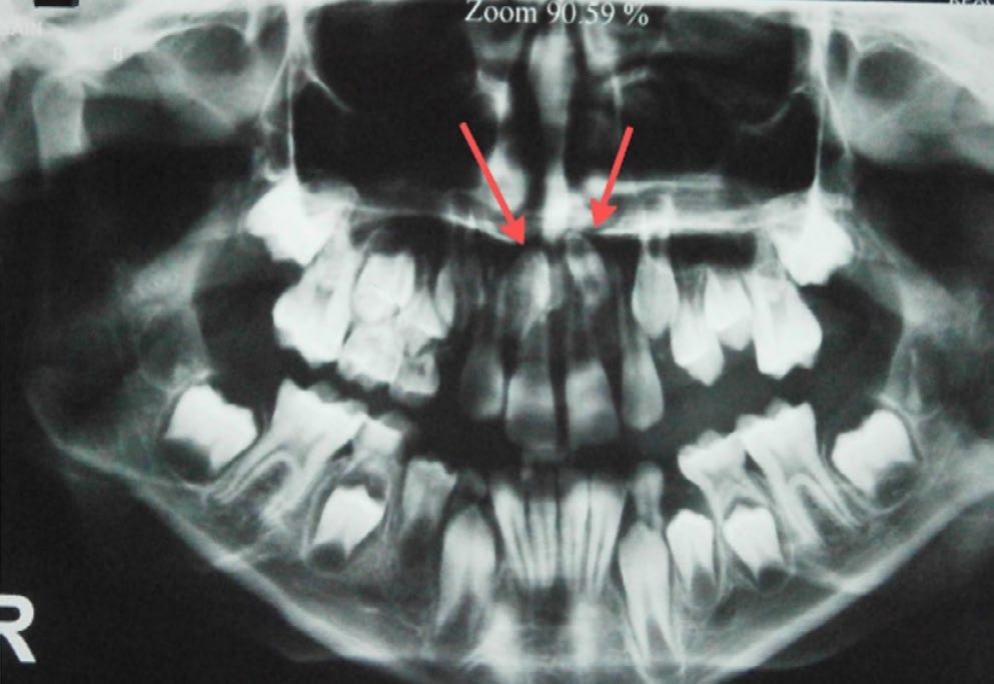
Panoramic radiograph showing deeply impacted supernumerary teeth in the anterior maxilla

**FIGURE 2:**
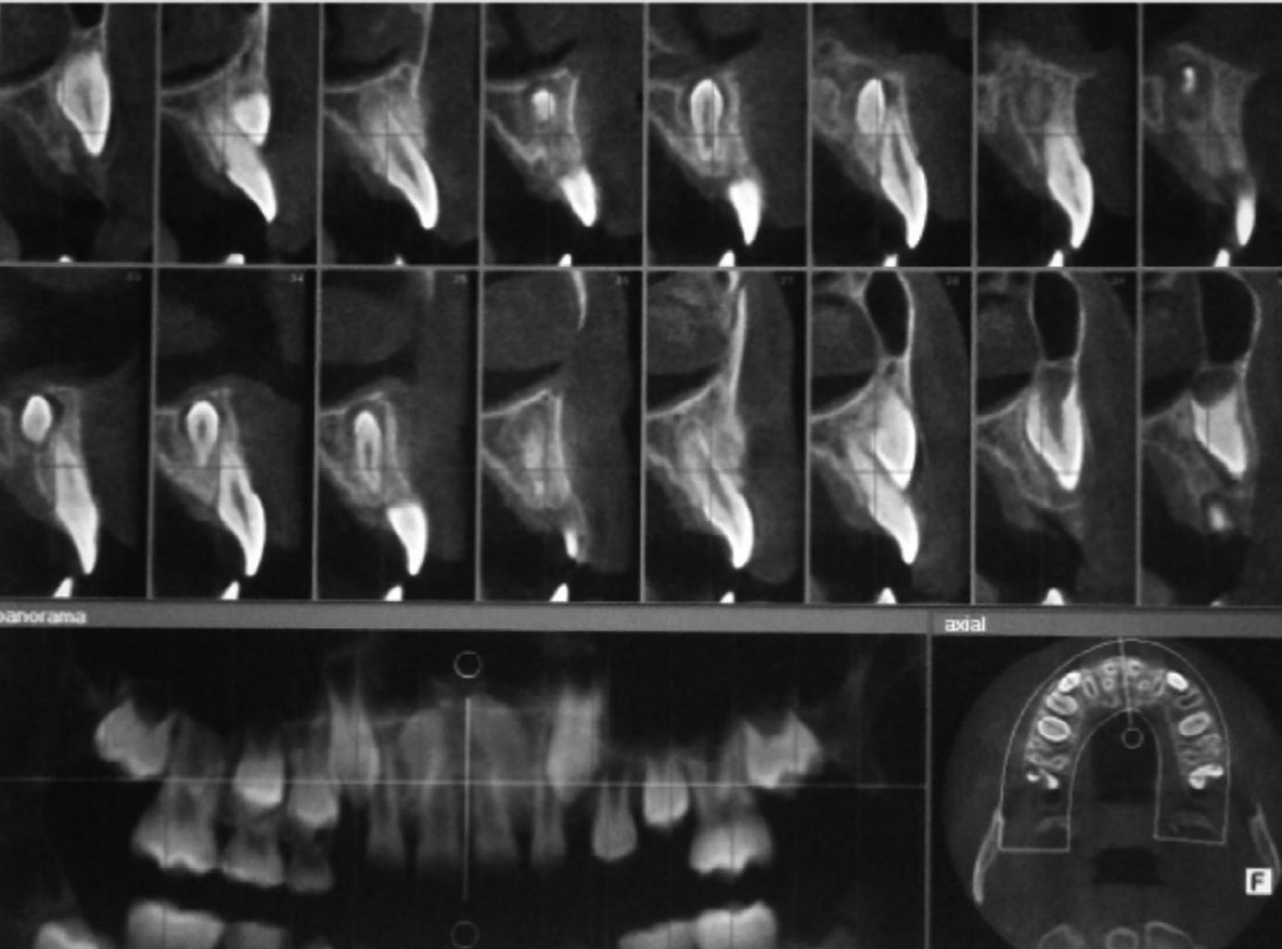
Sagittal CBCT images showing deeply inverted position of both mesiodens and their proximity to the adjacent root of permanent incisors

In December 2019, a 9‐year‐old Iranian boy was referred to our department (Oral and Maxillofacial Surgery Department of school of Dentistry, xxxxxxxx University of Medical Sciences) with a chief complaint of two bilateral deeply impacted mesiodens. The patient had first presented to the Department of Pediatric Dentistry at School of Dentistry of xxxxxxx University of Medical Sciences for a routine dental checkup. The patient's past medical history was unremarkable, and there was no familial history in terms of mesiodens or other forms of supernumerary teeth. Intraoral examination showed normal dentition, and no abnormalities were found in occlusion or tooth alignment. A panoramic radiograph was obtained, which showed two bilateral deeply impacted mesiodens in an inverted position (Figure 1). A cone‐beam computed tomography (CBCT) was requested to further assess the details regarding the exact position of mesiodens relative to the nasal floor and the adjacent permanent teeth. Sagittal CBCT scans showed deeply inverted position of both mesiodens and their proximity to the roots of the adjacent permanent incisors. The crowns of the two mesiodens had been covered with a very thin layer of the nasal floor. Both mesiodens were located in the anterior region of the nasal floor and close to the anterior nasal spine (ANS) and the piriform aperture (Figure 2). The distance between the crowns of the two mesiodens that were located bilaterally at both sides of the nasal septum and ANS is an important variable that determines the required amount of nasal floor dissection.

Written informed consent was obtained from the patient and his parents prior to the surgical procedure. Also, the patient and his parents consented to the publication of this report.

## 3 SURGICAL TECHNIQUE

**FIGURE 3:**
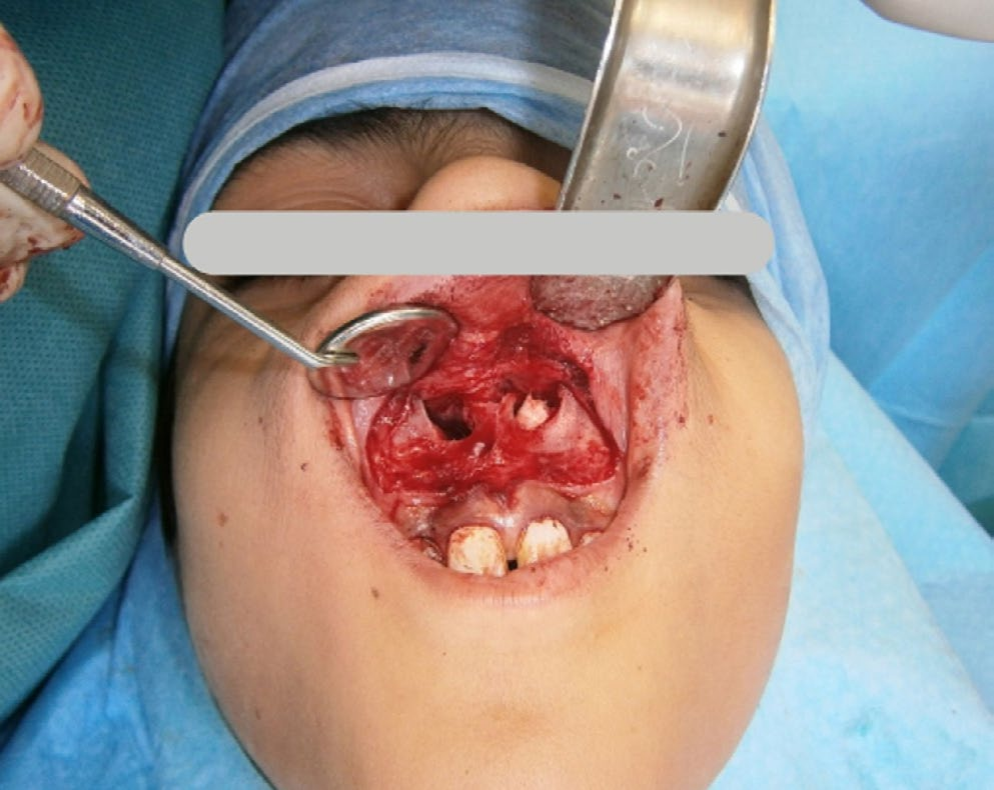
Uncovered impacted mesiodens crown during extraction from the nasal cavity side

The patient was requested to rinse his mouth with 2% chlorhexidine mouthwash (Iran‐Najo) for 20 s, and then, standard surgical prepping and draping for oral surgery under local anesthesia were performed. After topical application of 20% benzocaine gel (Master‐Dent, Dentonics) for topical anesthesia of the injection sites, local anesthesia was administered using lidocaine plus 1:80000 epinephrine (Persocaine‐E, DarouPakhsh). The incisive and bilateral greater palatine nerve blocks were administered for total nasal floor anesthesia. The buccal vestibular infiltration anesthesia was also administered to ensure a painless procedure. Next, a full mucoperiosteal vestibular scalloped incision was made right above the mucogingival junction (1–2 millimeters superior) from the lateral incisor of the right side to the lateral incisor of the left side. Two small releasing incisions were made at both horizontal ends of the first incision posterosuperiorly for easier dissection and exposure, considering a “V” shaped morphology around the upper lip frenulum. A mucoperiosteal flap was elevated, and the caudal margins of the piriform aperture and ANS were exposed with a periosteal elevator. Next, subperiosteal dissection of the nasal mucosa was performed to expose the impacted supernumerary teeth. The small amount of nasal floor bone above the mesiodens was removed by a rotary bur under saline irrigation, and the crowns of the two mesiodens were exposed (Figure 3). The piezosurgery device was also used for safer bone removal to avoid damage to the nasal mucosa and the adjacent structures, that is, the roots of the adjacent permanent teeth and the nasopalatine nerve. Use of piezosurgery to remove the small caudal bony part of the piriformis for better exposure is beneficial when the crown extends more posteriorly to the base of the nasal cavity. After elevating and extracting the mesiodens, the sockets and the wound area were rinsed with saline to eliminate debris. The wound was then closed with absorbable 4–0 vicryl suture (polyglactin 910, Ethicon) in one layer. The patient experienced no pain or discomfort during the surgery, and there were no postoperative complications such as nose bleeding or damage to the adjacent teeth.

## 4 DISCUSSION

Evidence shows that complications occur in approximately one‐half of patients with mesiodens. These complications may include delayed eruption of the adjacent permanent teeth usually central incisors, persistent midline diastema, root resorption of the adjacent teeth, cyst formation such as dentigerous cyst even in the nasal cavity, eruption into the nasal cavity, persistent rhinosinusitis, and pain.^3^, ^7^, ^8^ Different types of impactions and orientations of mesiodens require different surgical access and approaches. Deeply impacted inverted mesiodens with palatal position relative to the roots of the central incisors is traditionally extracted via a palatal or vestibular approach that obviously needs excessive bone removal and is associated with high risk of injury to the adjacent structures such as permanent teeth or their blood supply. Nasal mucosal perforation, and more difficult extraction because of the crown orientation, lack of a good grip for the elevator or forceps, and poor visibility are other problems encountered in use of traditional surgical approaches. The intraoral transnasal approach employed for our case provides much better visibility and has fewer complications with regard to traumatization of the adjacent structures.^5^, ^9^, ^10^, ^11^

Another important point is to perform the surgical intervention at a right time before the emergence of dental developmental problems or malocclusion. Thus, most patients are younger than 12 years of age at the time of surgical procedure. Also, the surgeon should ensure a painless procedure and patient comfort. For this reason, most clinicians prefer general anesthesia or deep sedation, which require hospitalization and administration of anesthetic agents. This can cause financial problems for the patients. Also, deep sedation may have some adverse effects such as snoring, crying, vomiting, intravenous site pain, and aspiration.^6^, ^12^ Contrary to the reports that proposed general anesthesia or had recommendations regarding sedation,^9^, ^12^ our experience showed that our suggested modified nasal approach under local anesthesia created a painless and comfortable experience for the patient. The most important issue here is detailed and thorough evaluation of the position of mesiodens that leads to thoughtful planning of the surgical approach and estimation of the required amount of nasal floor deflection and the level of discomfort it may cause during local anesthesia, which was made perfectly clear before surgery in our study by using CBCT.

## CONFLICT OF INTEREST

None.

## AUTHOR CONTRIBUTIONS

Reza Sharifi: Idea, hypothesis, surgical procedure, and proofread the manuscript. Shervin Shafiei: Search, screening, data extraction, quality assessment, writing of introduction and discussion, review of articles. Hamidreza Moslemi: Search, screening, data extraction, quality assessment, writing of method and materials (technical notes). Meysam Mohammadi Khah: Data extraction, quality assessment, surgical procedure, proofread the manuscript, and corresponding author.

## ETHICAL APPROVAL

This study was performed according to the principles outlined by the World Medical Association's Declaration of Helsinki on experimentation involving human subjects, as revised in 2000 and has been approved by the ethics committee of the Tehran University of Medical Sciences.

## CONSENT

Written informed consent was obtained from the patient and his parents prior to the surgical procedure. Also, the patient and his parents consented to the publication of this report.

## Data Availability

The data that support the findings of this study are available from the corresponding author upon reasonable request.
